# Salt and surfactant coated filters with antiviral properties and low pressure drop for prospective SARS-CoV2 applications

**DOI:** 10.1038/s41598-022-15203-9

**Published:** 2022-07-07

**Authors:** Milad Ebadi, Claire McCague, Ophelia Vallee, Patrick K. Taylor, Amy H. Y. Lee, Majid Bahrami

**Affiliations:** 1 Laboratory for Alternative Energy Conversion (LAEC), School of Mechatronic Systems Engineering, Simon Fraser University, Surrey, BC V3T 0A3 Canada; 2 School of Health Science, British Columbia Institute of Technology, SW 3, White Ave, Burnaby, BC V5G 3H2 Canada; 3 Department of Molecular Biology and Biochemistry, Simon Fraser University, 8888 University Drive, Burnaby, B.C V5A 1S6 Canada

**Keywords:** Biological techniques, Health care, Engineering, Biomedical engineering, Chemical engineering, Mechanical engineering

## Abstract

The COVID-19 pandemic motivated research on antiviral filtration used in personal protective equipment and HVAC systems. In this research, three coating compositions of NaCl, Tween 20 surfactant, and NaCl-Tween 20 were examined on polypropylene spun-bond filters. The pressure drop, coverage, and crystal size of the coating methods and compositions were measured. Also, in vitro plaque assays of the Phi6 Bacteriophage on *Pseudomonas syringae* as a simulation of an enveloped respiratory virus was performed to investigate the antiviral properties of the coating. NaCl and NaCl-Tween 20 increased the pressure drop in the range of 40–50 Pa for a loading of 5 mg/cm^2^. Tween 20 has shown an impact on the pressure drop as low as 10 Pa and made the filter surface more hydrophilic which kept the virus droplets on the surface. The NaCl-Tween 20 coated samples could inactivate 10^8^ plaque forming units (PFU) of virus in two hours of incubation. Tween 20 coated filters with loading as low as 0.2 mg/cm^2^ reduced the activity of 10^8^ PFU of virus from 10^9^ to 10^2^ PFU/mL after 2 h of incubation. NaCl-coated samples with a salt loading of 15 mg/cm^2^ could not have antiviral properties higher than reducing the viral activity from 10^9^ to 10^5^ PFU/mL in 4 h of incubation.

## Introduction

Filters can remove particles, microorganisms, and viruses from the air and make the air clean; hence, there will be a high concentration of microorganisms and viruses inside them and they can be a point of contamination themselves^1^. Zhinguing et al.^2^ examined the face masks of the medical staff for contamination and concluded that the most dominant bacteria that contaminated the inside and outside areas of the used masks were the *Staphylococcus* spp. and *Pseudomonas* spp. Al-abdalali et al.^1^ cultured swabbed samples from air-conditioning system ducts and found them to be contaminated by six different types of bacteria and five fungus species. Antiviral and antibacterial filtration is proposed as a solution to reduce the risk of cross-infection and the spreading of microorganisms, as well as to reduce the demand for single-use filters. However, there are some challenges, including reliability, breathability, durability, reproducibility, and price^1,3–9^.

KCl, K_2_SO_4_^10^, grapefruit seed extract^3^, iodine^11^, citric acid^12^, and natural table salt (NaCl) have been applied to filters for their antiviral properties. NaCl, with its high availability and historical disinfection properties, is one of the materials that was recently reported to have antiviral properties on filters^7,8,10,13,14^. Quan et al.^13^ first used a NaCl solution as a coating material for polypropylene fibers with the help of Tween 20 as a surfactant to overcome the hydrophobic properties of the filters. They reported that 5 mg/cm^2^ of the coating composition can eliminate the influenza virus after 60 min. To investigate the viral activity change, they used the hemagglutinin activity change of the influenza virus and the mice infection test in a closed container. Rubino et al.^10^ used the same method for NaCl, KCl, and K_2_SO_4_ salts on large pore filters and concluded the same results. To investigate the antibacterial properties, they used different amounts of NaCl coated on the filters to see the stability of *K. pneumonia* aerosols. Rubino et al.^8^, performed X-ray diffraction (XRD), Energy Dispersive X-ray Analysis (EDX), and microscopy analyses to investigate the main reasons for the antiviral properties of NaCl. Based on their results, the recrystallization of salt after several wetting and drying cycles causes the microorganisms to lyse, leading to their inactivation. Antibacterial properties were measured using both in vivo and in vitro stability analyses of *K. pneumoniae* and the influenza virus. In all of their experiments, the Tween 20 surfactant was used to improve the coating and drop-cast was used as the coating method. However, Tween 20 has detergent properties and can inactivate microorganisms even in concentrations as low as 0.05% v/v^15,16^.

There is limited research comparing salt coatings to salt and surfactant-coated filters in terms of coating coverage and antiviral properties. Moreover, there is insufficient information in the literature on the crystal size and its effect on pressure drop, although, it is one of the most important parameters of filters in practical usage. Adding a nano-dry-salt (NDS) is another method to add antiviral properties to filters. Park et al.^14^ used this method to show the antiviral properties of salt for the aerosolized coronavirus. They used a nebulization coating method to coat the polypropylene spun-bond filters with a salt loading as low as 3 mg/cm^2^. Based on their results, 14.5 × 10^9^ particles/cm^2^ can result in a reduction of the viral activity of the HCoV-229E virus. The presented method will have a minor effect on pressure drop and slight antiviral properties.

In this research, three methods of coating and nine compositions of salt and surfactant were examined. The average crystal size and coverage of the coatings were measured as a function of loading, and the benefits and drawbacks of each deposition method are reported. The pressure drop across uncoated and coated filters was also measured using the standard EN-14683^17^, and the effects of each coating method and loading were evaluated. The effectiveness of the filter against membrane viruses was measured using established plaque assays^18^ with the viability of the enveloped virus of Phi6 bacteriophage measured by the viral ability to infect the bacterial host *Pseudomonas syringae*^19^. The samples coated by nebulizer were subjected to a plaque assay activity measurement to evaluate the antiviral properties of the coating on the virus-loaded droplets. To examine the main cause of antiviral properties, three concentrations of NaCl, Tween 20, and NaCl- Tween 20 coated samples were subjected to plaque assay activity measurements using 10 µL micro pipette transferred droplets. Viruses transferred with micro pipette are more stable than aerosols for Phi6 in higher temperatures and relative humidity as a common condition for mask application^20,21^. Finally, a test was conducted to determine the efficacy of coated spun-bond layers to stop the penetration of active viruses through the whole filter.

## Results and discussion

### Salt coating

**Figure 1:**
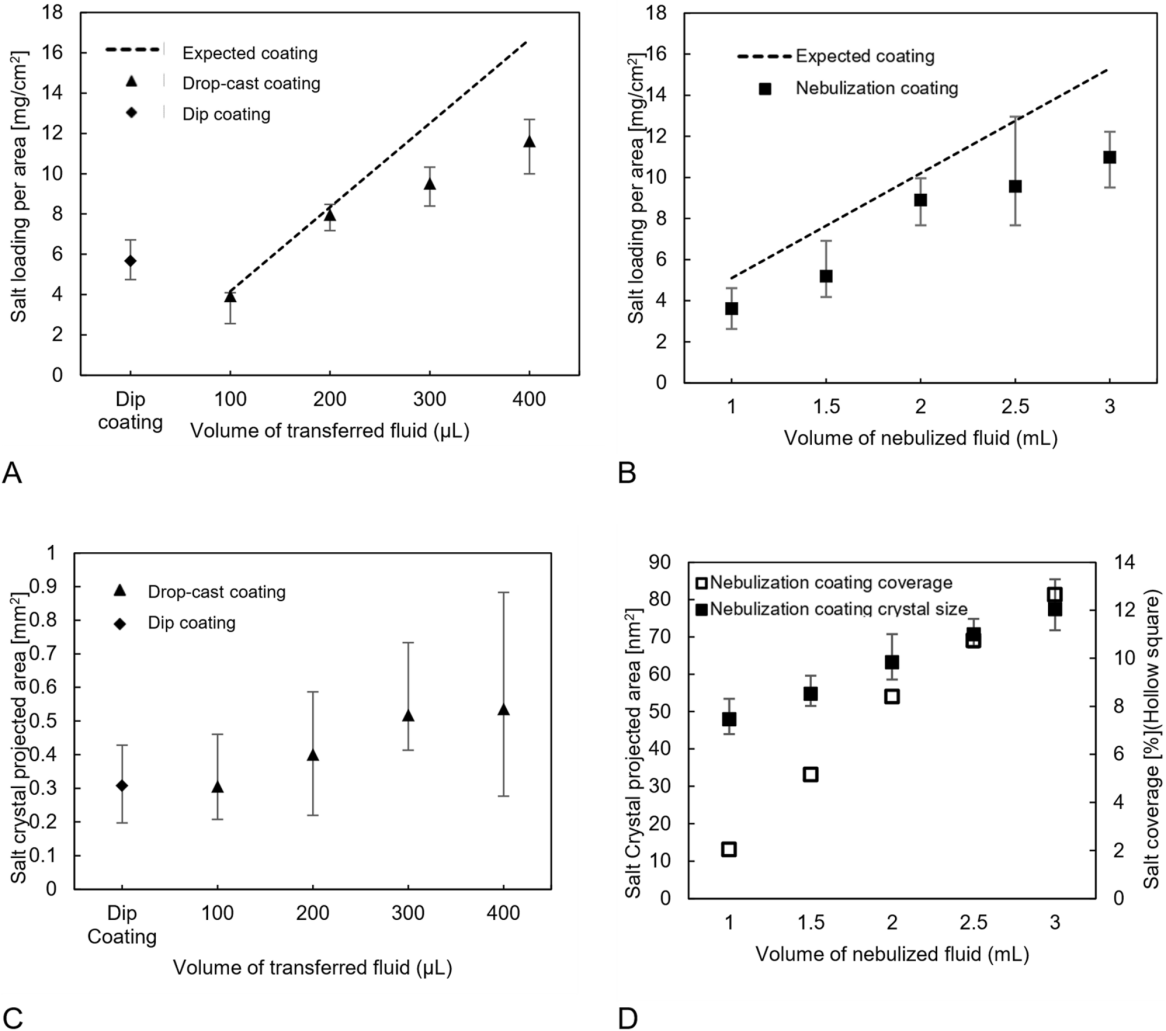
(**A**) Salt loading vs. transferred fluid for dip coating and drop-cast coating; (**B**) Salt loading vs. nebulized fluid for nebulized coating; (**C**) Salt crystal size vs. the volume of transferred fluid for dip coating and nebulized coating; and (**D**) Salt crystal size and coverage vs. the volume of nebulized fluid for samples coated by nebulization.

**Figure 2:**
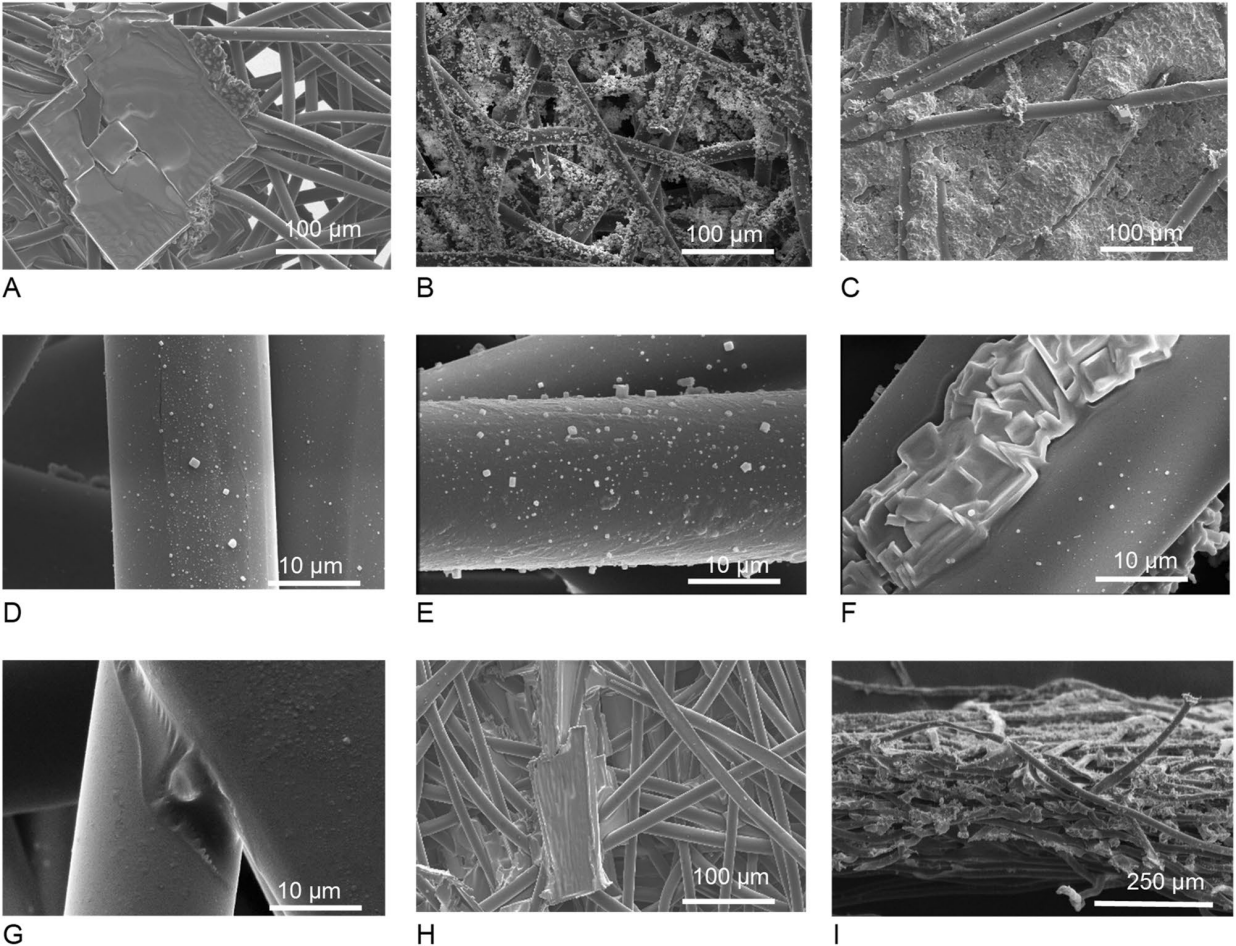
Crystal size and coverage (**A**) For a 5 mg/cm^2^ drop-cast; (**B**) a 5 mg/cm^2^ nebulized coating (**C**) an overloaded filter with 10 mg/cm^2^ of coating (**D**) 5 mg/cm^2^ of a NaCl-coated sample. Picture used for crystal size and coverage measurement; (**E**) 5 mg/cm^2^ of a NaCl-Tween 20 (50:1)-coated sample used for crystal size measurement; (**F**) The effect of surfactant in reducing surface tension and droplet spreading and salt recrystallization on 5 mg/cm^2^ of a NaCl-Tween 20 (50:1)-coated filter; (**G**) A surfactant droplet and the capillary effect resulted from hydrophilicity; (**H**) The large crystal resulted from 10 mg/cm^2^ of a NaCl-Tween 20-coated which caused the choking of the air path and an increase in the pressure drop; and (**I**)The coating of NaCl-Tween 20 deep within the filter*.*

The results for dip, drop-cast, and nebulization coating are presented in Figs. 1 and 2. Based on previous studies, the coating aims for 5 to 15 mg/cm^2^ of NaCl^8,10,13,22^. The salt loading for the dip-coating method is presented in Fig. 1A. For dip-coating method, the loading is a function of fluid viscosity, surface tension, and substrate; therefore, while reproducible salt loading is readily achieved, using saturated salt solution limited the loading to 5 mg/cm^2^. Optical crystal size measurements are presented in Fig. 1C. The crystals size, using dip-coating method, was in the range of 200–500 µm which is large enough to block the airstream and render a part of the filter a dead zone.

The drop-cast coating results of salt loading for various volumes of solution are depicted in Fig. 1A. For lower volumes of fluid, the loading is close to the theoretical amount because the transferred fluid was retained by the filter. When the salt solution volume was increased, leakage of salt solution from the filter decreased the loading relative to the maximum calculated amount that could be deposited by the solution. As shown in Fig. 1C, Increasing the volume of transferred solution from 100 to 400 µL of the affected the mean crystal size to change from 300–500 µL but it will increase the variation of the crystal size. Figure 2A shows the large crystals resulting from the drop-cast coating, which are the same as for the dip coating. This large crystal size can increase the pressure drop by blocking the air path.

The other coating method examined was the aerosolization coating. This method can produce fine crystals on the order of nanometers. Aerosolization was challenging and led to the large variation in coating, especially in large salt loadings, because it is highly related to the filter’s performance in capturing nanoparticles. In terms of crystal size, Fig. 1D represents the crystal size at the left axis and the coating coverage at the right axis for different volumes of solution on the innermost surface of the filters. The coating coverage is based on the total covered area and by increasing the volume of the salt solution, the coverage will increase, as well as the crystal size. This is because the fibers’ surface will get wet and this wetness will coagulate the droplets and increase the particle size. The low surface coverage is because the coverage was measured from the innermost layer of the filter as the minimum coverage. Figure 2I shows the coverage of the coating in the thickness of the filter. The coating is almost constant in thickness with a slight reduction for the inner layers.

The effect of the coating solution is shown in Fig. 2D–I. Figure 2D is the coating resulting from the NaCl solution and Fig. 2E,F are the coatings resulting from the NaCl-Tween 20 coating solution. As can be seen, the NaCl-Tween 20 solution shows a uniform coating layer around the filter, while the NaCl coating has discreet crystals covering the fiber’s surface. Because the surfactant can reduce the fluid’s surface tension in locations where fibers pass closely through each other, a cluster of coating material will be made due to the capillary effect, as illustrated in Fig. 2F for NaCl-Tween 20 and Fig. 2G,H for Tween 20. However, this effect was not noticed for the NaCl-coated samples that show this was due to the surfactant.

To investigate the effect of the surfactant alone, fiber filters were coated with 0.2 mg/cm^2^ of loading. Figure 2G shows nanometric splashes of surfactant coated on the fibers and a cluster of coating located at the locations where fibers are close to each other.

### Effect of coating on pressure drop

**Figure 3:**
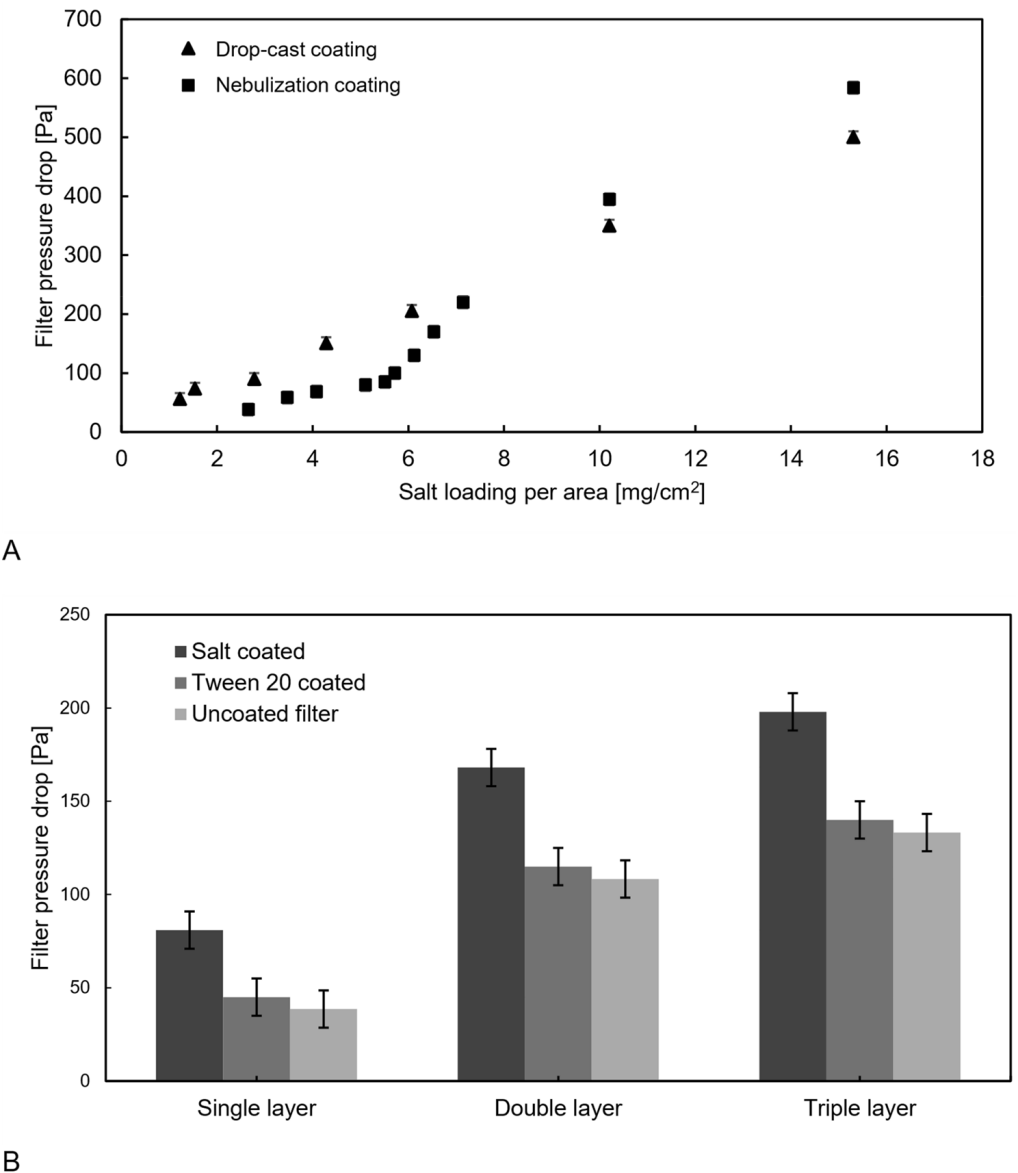
(**A**) Pressure drop vs. salt loading for drop-cast coating and nebulization coating; rapid increase in pressure drop after 6 mg/cm^2^ of loading for nebulization coating; (**B**)The effect of multilayer filters on the pressure drop for NaCl-Tween 20 and NaCl in comparison with uncoated filter. The variation in pressure drop between Tween 20 coated and uncoated filters is less than the uncertainty of the experiment.

Based on ES-14683, the pressure drop of the samples was measured. As can be seen in Fig. 3A, for the lower loading, the nebulized coating has a lower pressure drop but it will rapidly increase near 6 mg/cm^2^ of coating and gets higher than the drop-cast coating in higher loading amounts.

Figure 2B shows this phenomenon. Lower coating loading keeps the pores of the filter open while the salt coats the fibers thoroughly. Higher loading in the filter results in the forming of a cluster of salt on the foremost layer of the filter and a rapid increase in pressure drop (Fig. 2C). Although the aerosolization coating can generate fine crystals, the formation of particle clusters can increase the pressure drop dramatically, Fig. 2C. This cluster formed because of the deposition of particles in the foremost layer of the filter to the airstream. To prevent formation of cluster, 5 mg/cm^2^ of salt loading was found to be the optimum amount of loading for this type of filter to reach the appropriate coverage and acceptable pressure drop, see Fig. 2B.

For drop-cast coating, district crystals will make dead zones and result in higher pressure drops for lower loading, while due to the higher density of the district crystals than nebulized crystals which are fluffy, the pressure drop will be lower than for nebulized coating samples. The crystals are generally smaller in lower loading and larger for higher loading. This causes the constant trend in pressure drop increase.

As depicted in Fig. 3B, adding multiple layers of 5 mg/cm^2^ of NaCl-loaded layers has a lower effect on pressure drop than adding the same amount of coating on a single layer. This is due to more uniform particle dispersion and lower cluster formation in the flow.

Knowing the effect of salt loading on pressure drop, it is worth observing the pressure drop of surfactant-loaded filters. As can be seen in Fig. 2B, adding the surfactant has some minor effects on pressure drop and shifts the pressure drop to less than 10 Pa, which is negligible and in the range of uncertainty. Thus, Tween 20 coated samples can be a better alternative for the NaCl coating if they have enough antiviral activity.

### Biological validation of antiviral properties

#### Method validation

To determine whether the filters and coating were effective against membrane viruses, we performed a well-established plaque assay using the bacteriophage Phi6 and *Pseudomonas syringae*. We performed control experiments to ensure (1) that the washing buffer did not impact viral or bacterial stability and (2) any reduction in virus numbers as measured by plaque forming units we observed were not due to inter-experimental differences but filter coatings.

#### Washing method validation

The coating materials in filters are minerals soluble in water, ideally, all the coating material will be transferred to the solution during the washing of viruses, and Phi6 will be present in all of the stages of the experiment after washing for quantification. Thus, there must be a method to evaluate the effect of the washing solution on the viral and host bacterial viability. To do so, one experiment was performed to evaluate the bacterial stability and three to probe virus stability.

##### Bacterial stability

**Figure 4:**
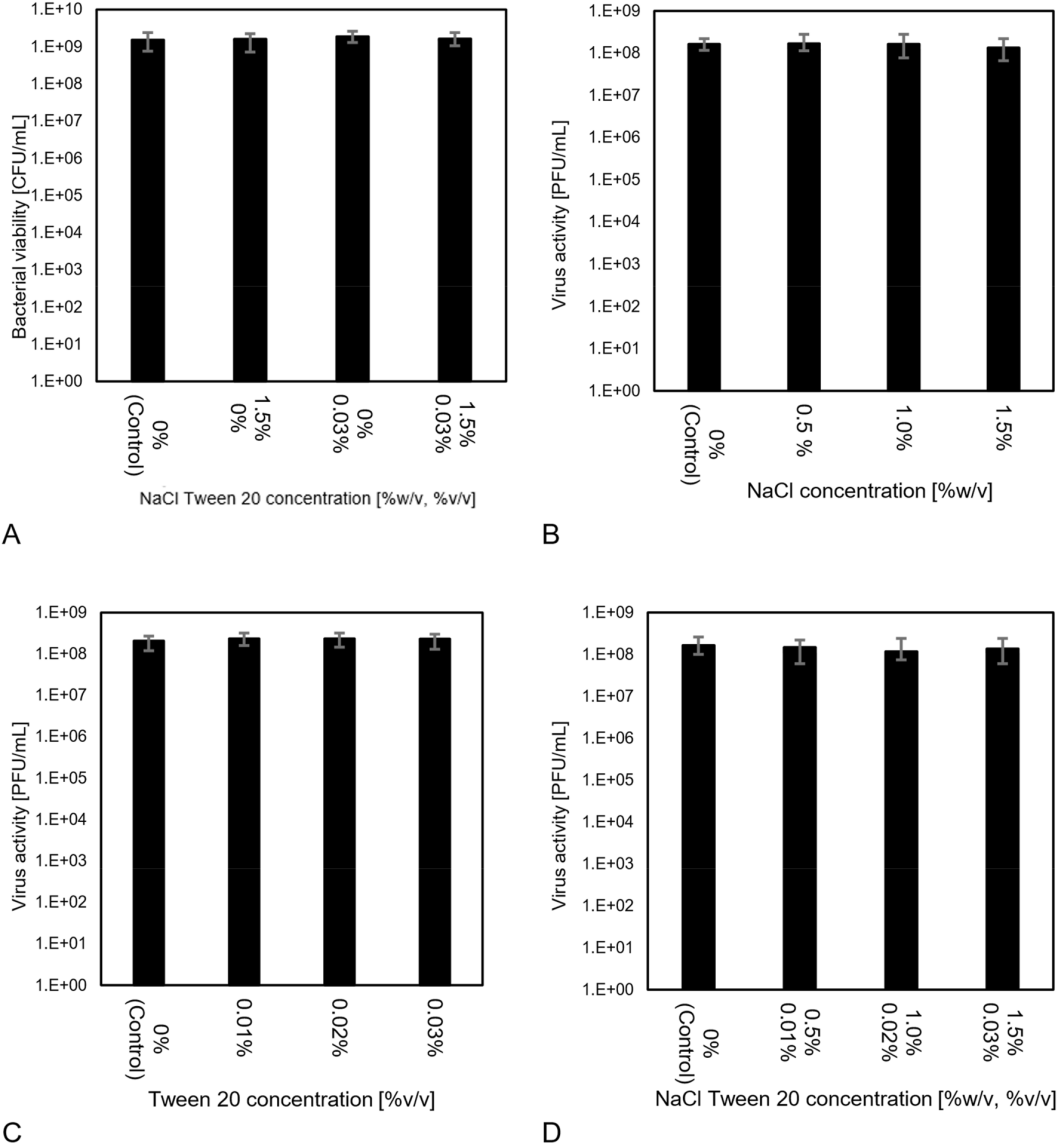
Verification of Phi6 and bacterial stability in the virus extraction solution consisting of King Broth (KB) and active ingredients that would be washed from sample filters during extraction. (**A**) Bacterial viability in washing solution containing 0% to 1.5% w/v NaCl and 0% to 0.03% v/v Tween 20; (**B**) Viral stability in washing solution containing 0% to 1.5% w/v NaCl. (**C**) Viral stability in washing solution containing 0% to 0.03% v/v Tween 20; (**D**) Viral stability in washing solution containing 0% to 1.5% w/v NaCl and 0% to 0.03% v/v Tween 20.

The bacterial stability method is presented in Sect. 7.8. The experiments were performed with the highest concentrations of each coating composition to simulate the most extreme conditions. As shown in Fig. 4A, the bacterial viability remained almost constant (10^9^ CFU/mL) for all three coating compositions. The effect of the change in the coating material on bacterial viability was negligible (F-value 0.172; P-value 0.912). This is because the maximum surfactant and salt concentrations in the washing solutions, (0.03% v/v and 0.15% w/v, respectively), were low relative to the known threshold for antibacterial activity^15^.

##### Viral stability

Virus stability test method is presented in Sect. 7.8. Three concentrations of salt, surfactant, and the combination of salt and surfactant were examined to determine if the washing procedure affected the virus activity. As shown in Fig. 4B, there is a slight reduction (F-value 1.77; P-value 0.167) in activity during the washing of the NaCl-coated filters but this reduction is not considerable and is lower than experimental uncertainties. For Tween 20, Fig. 4C, (F-value 1.92; P-value 0.142). The higher F-value shows the higher dependence of the solution on the surfactant concentration; however, this is a lower critical value and still can be neglected. For NaCl- Tween 20 (Fig. 4D), the F-value is 1.94 and the P-value is 0.14. The small effect of Tween 20 on the viral and bacterial activity in the washing solution is the result of the low concentration of the surfactant and the high concentration of protein in the culture media. The protein in the washing solution prevents micelle formation which reduces the detergent properties of the surfactant solution^23,24^. The short time of susceptibility (almost 1 h) and high viral activity also play key roles in reducing the effect of the surfactant in washing procedures.

##### Time stability

**Figure 5:**
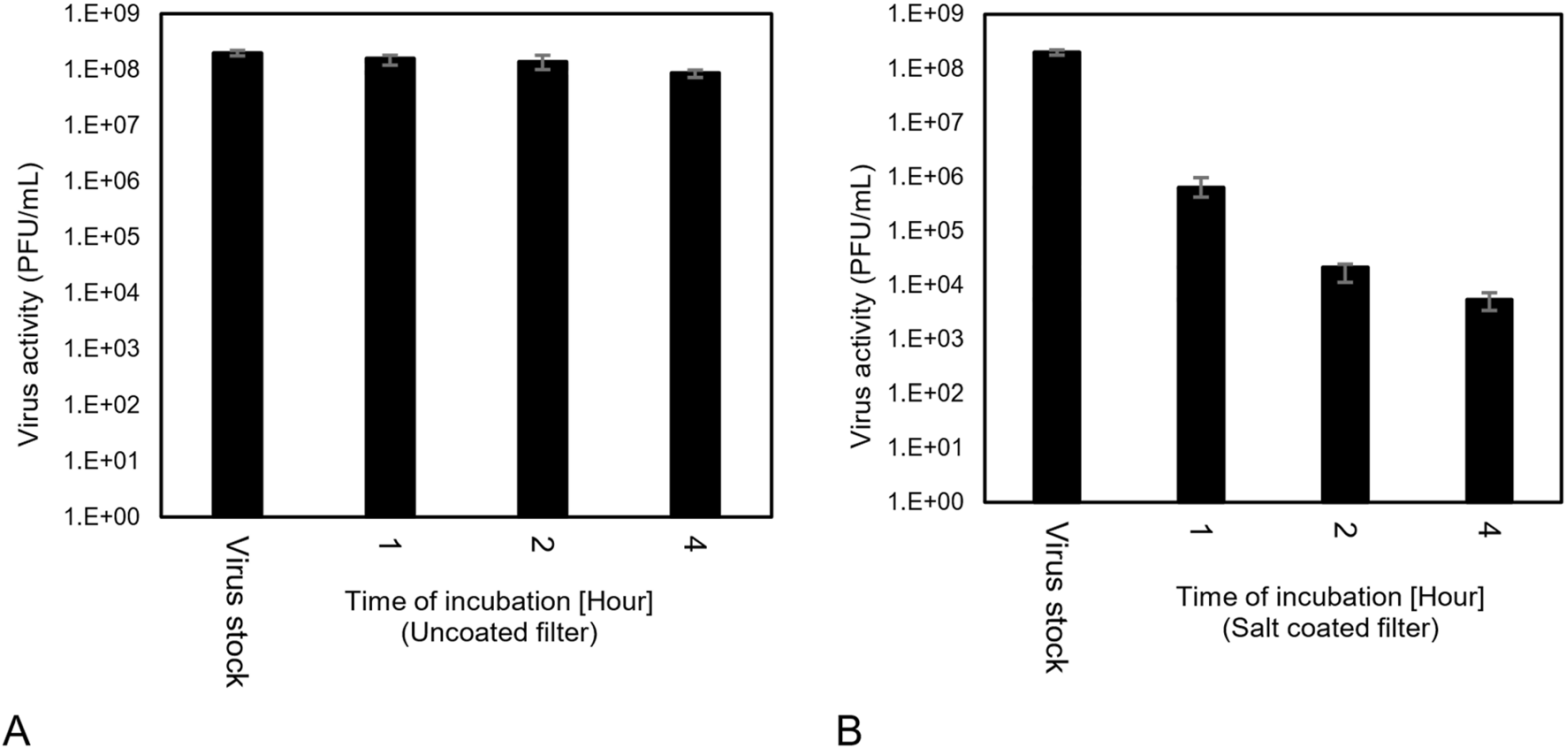
The time stability of virus on fibrous filters: (**A**) Uncoated filter; (F-value 26; P-value 4 × 10^–7^) and (**B**) a 5 mg/cm^2^ NaCl-coated filter. Virus is stable on surface of the filter and salt will reduce the activity during time (F-value 1985; P-value 6.5 × 10^–25^).

Virus deactivation requires exposure time on the surface^8,10,13^. To evaluate the effect of the exposure time on the virus activity, the virus time stability on the uncoated and NaCl-coated filters was examined. The viral activity of Phi6 is known to decline during incubation and particularly for incubation time more than 5 h after transferring to the filter surface^7,8,21,25–27^. In this research, the virus remained relatively stable when 10 µL droplets (10^8^–10^9^ PFU/mL) were dispersed on the filters or glass (for control) and was chosen to be the volume of droplet to be transferred to the filter for the next experiments. As shown in Fig. 5A, the viral activity in droplets decays slightly from 2 × 10^8^ to 8.8 × 10^7^ PFU/mL during the 4 h of incubation (F-value 26; P-value 4 × 10^–7^). This indicates that the viral activity decay is due to the incubation time. However, as shown in Fig. 5B, for NaCl-coated filters, the viral activity will reduce from 2 × 10^8^ to 10^4^ PFU/mL, indicating that the NaCl coating has some antiviral effects but does not significantly deactivate the virus (F-value 1985; P-value 6.5 × 10^–25^). Based on the results, 2 h of incubation was chosen to be the time for incubation. Comparison of the NaCl-coated filters with the Park’s^14^ research shows both the HCoV virus and the Phi6 bacteriophage reacted in the same way in the presence of NaCl nanoparticles with the same fraction of reduction of activity for a short exposure of the virus with the coated filter.

#### Antiviral properties of a coated filter

The effective salt loading amount in previous works is on the order of 5–20 mg/cm^2^^7,8,10,13^. As discussed in Sect. 5.2, the coating load should be kept lower than 5 mg/cm^2^ to keep the pressure drop in an acceptable range. Therefore, multiple layers of filters with a salt loading of 5 mg/cm^2^ were used in the experiments which needed higher salt loading. To find the optimum antiviral material for coating, NaCl, Tween 20, NaCl-Tween 20 coated filters with three loading concentrations were examined in biological validations. Simultaneous control experiments were performed on uncoated filters to verify the results.

**Figure 6:**
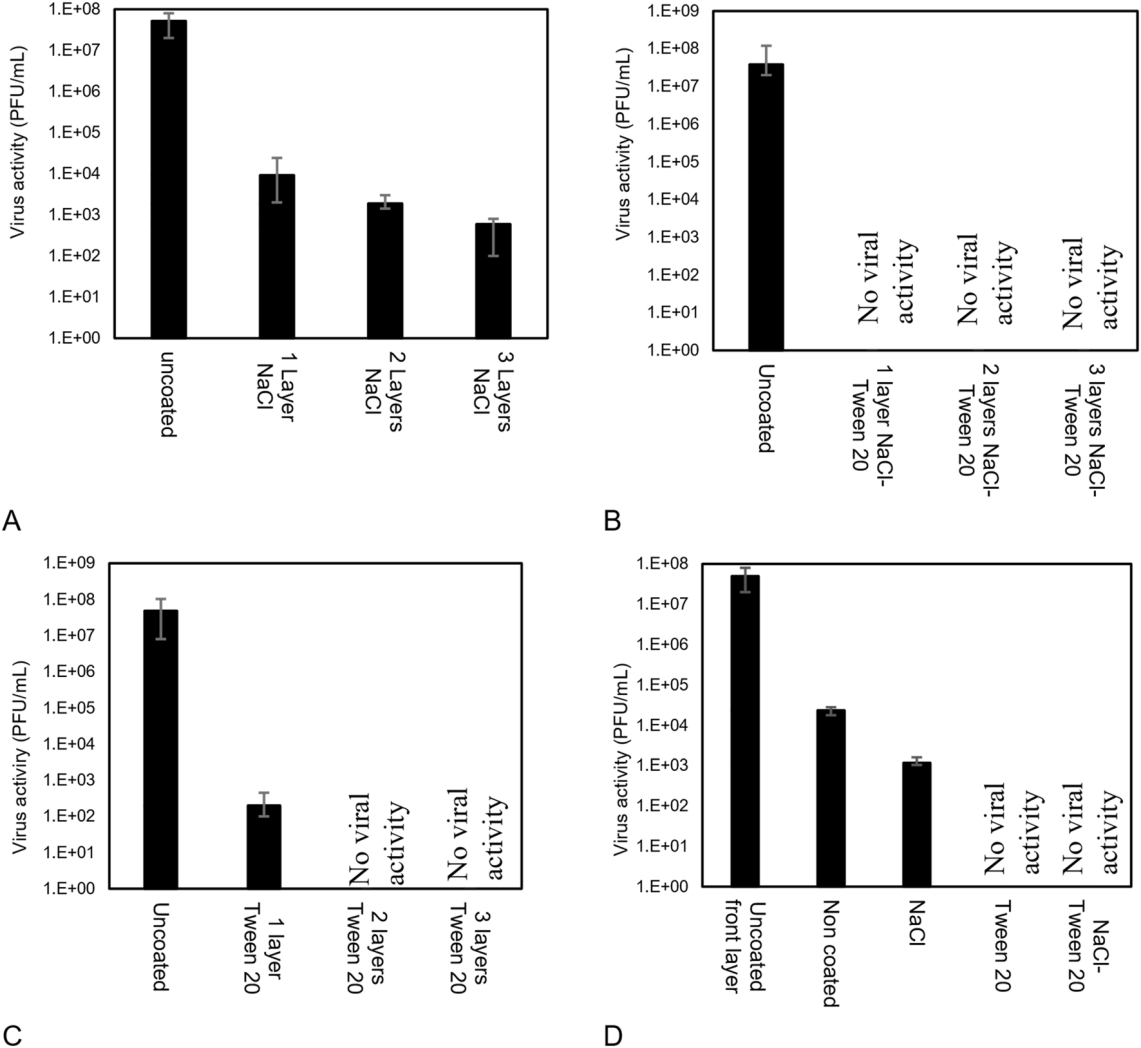
(**A**) The antiviral properties of 5 mg/cm^2^ of NaCl-coated filters, effect of layers; (F-value 84.8; P-value 2.57 × 10^–15^) (**B**) The antiviral properties of 5 mg/cm^2^ of NaCl-Tween 20 (50:1) coated filters, effect of coating loading and layers; (Uncoated: Average = 5.0 × 10^7^ PFU/mL, σ = 3.2 × 10^7^ PFU/mL, 1-Layer, 2 Layers and 3 Layers was below detection limit) (**C**) The antiviral properties of 0.1 mg/cm^2^ of Tween 20 coated filters, effect of layers; (Uncoated: Average = 4.03 × 10^7^ PFU/mL σ = 1.7 × 10^7^ PFU/mL, 1 Layer Tween20: Average = 1.60 × 10^2^ PFU/mL, σ = 4.2 × 10^1^ PFU/mL (T(10) = 43.6 P-value = 8.2 × 10^–7^) 2 Layers and 3 Layers was below detection limit) and D) The penetration of the active virus(Uncoated front layer: Average = 5.03 × 10^7^ PFU/mL, σ = 1.5 × 10^7^ PFU/mL, non-coated N95: Average = 2.4 × 10^4^ PFU/mL σ = 2 × 10^2^ PFU/mL, N95 sublayer of NaCl: Average = 1.1 × 10^3^ PFU/mL, σ = 2.3 × 10^2^ PFU/mL, NaCl vs N95 sublayer significance (T(10) = 43.6 P-value = 8.2 × 10^–7^ ), Tween20 and NaCl- Tween20 was below detection limit ).

As shown in Fig. 6A, the addition of more layers of NaCl-coated filters will increase the inactivation rate (F-value 84.8; P-value 2.57 × 10^–15^); however, the inactivation is incomplete. Therefore, either a higher loading of NaCl or an alternative coating composition should be used as an antiviral agent.

Adding 0.1 mg/cm^2^ of Tween 20 (per filter) to the NaCl coating increased the antiviral activity, such that, a single filter layer eliminated the virus with the activity of 5 × 10^7^ PFU/mL, Fig. 6B. The coated filters had viral activity lower than detection limit. The surfactant has detergent properties^15^ and can lyse microorganisms and interrupt their activity. The presence of surfactants on the filter increases their hydrophilicity such that virus contacting solution is wicked into the filters which also improves the antiviral activity of the coated filter. The hydrophilic properties of the fibers after being susceptible to the surfactant, made the virus droplets more susceptible to the salt particles and improved the antiviral activity.

Figure 6C shows, the filters coated with surfactant. Single layer surfactant coating could reduce the viral activity from 4 × 10^7^ to 1.6 × 10^2^ PFU/mL (T(10) = 43.6 P-value = 8.2 × 10^–7^) and 2 Layers and 3 Layers of coated filters had viral activity lower than detection limit. The surfactant-coated filters, also have considerable antiviral properties and are even greater than for the NaCl-coated filters which shows that the main cause for the antiviral property of the NaCl-Tween 20 coated filters is the surfactant detergent properties, and adding the NaCl can enhance the antiviral properties of the filters. The high antiviral properties of Tween 20 show that this material can be an effective antiviral agent to be used on the surface of filters. Coating concentration of 0.1 mg/cm^2^ is low, and a concentration of 0.2 mg/cm^2^ is needed to achieve the maximum antiviral activity.

##### Penetration of active virus

To explore the antiviral effect of different coatings and their ability to prevent penetration of active virus, a layer of the coated filter was placed on a layer of an N95 filter and the same procedure was performed to evaluate the viral activity for both coated filter and sublayer filter. As virus-containing solution (King Broth, KB) dries it leaves a light-yellow stain on the samples, which confirmed that the droplets penetrated through the coated filters to the uncoated sublayer. As shown in Fig. 6D. The viral activity of the penetrated virus from the uncoated filter is in the range of 10^5^ PFU/mL, while the activity is 10^4^ PFU/mL for the NaCl-coated filters (T(10) = 25.7, P-value = 2.8 × 10^–9^) and there was not any measurable viral activity in the sublayer of the Tween 20 and NaCl-Tween 20 coated filters. This experiment shows that the Tween 20 loaded coatings not only deactivate the virus on the surface of the filter, but also it can prevent the penetration of active viruses into the filter too.

### Discussion on Tween 20 antiviral properties

As mentioned in Sect. 5.3.2, the virus was delivered to the filters as 10 µL droplets. For the uncoated and NaCl coated filters due to hydrophobic properties of the surface, each droplet remained separated from the others until it evaporated in the incubator. However, due to lower hydrophobic properties in the Tween 20 coated filters, the droplets will be wicked into the filter and this phenomenon can lead to a high concentration of Tween 20 in the droplets. The total volume of the delivered virus solution was 50–250 µL and considering the 0.1–0.2 mg/cm^2^ of Tween 20 coated on the filter and 5 cm^2^ of total filter area, the regional concentration of Tween 20 in the droplet will be 0.2–2% v/v which is high enough to deactivate several types of viruses even when exposure is less than one minute^28^. The volume of the washing solution used on the filters is such that the concentration of Tween 20 is below 0.02% v/v which is close to critical micelle concentration and the antiviral activity is lower due to low concentration of micelles (5.3.2.2); also, the presence of the protein in the washing solution will reduce the potential for formation of micelles and reduces the effect of surfactant even more. This design of washing procedure is to minimize the effect of any treatment on the viruses and bacteria extracted from the filters after washing from the filters. As a result, the antiviral activity can be attributed to the filters coated by antiviral agents.

To compare the result of this research with the aerosolized virus-containing droplets, based on the filter’s characteristics, the total surface area of the fiber surfaces will be ⁓12 cm^2^ which leads to an average of 0.5 µm of a Tween 20 coating thickness in an ideal situation. Considering a virus containing droplet has a diameter in the range of 0.3–3 µm is captured by the filter, the approximate concentration of the coating by the droplet will be in the range of 5–50% v/v which is much higher than the minimum required concentration of the surfactant for deactivation and leads to rapid deactivation of viruses.

The low-pressure drop effect of the coating along with high antiviral activity and low material consumption, make Tween 20 a good coating for face mask applications. Also, due to the liquid nature of the Tween 20, it sticks to the surface better. Conversely, NaCl-coated filters have a high pressure drop effect and can produce microcrystals that can penetrate the human respiratory system.

## Conclusion

In this study, the antiviral properties and pressure drop of polypropylene filters coated with NaCl, Tween 20 surfactant, and NaCl-Tween 20 (50:1) were evaluated. Three coating methods were examined. The coating quality, crystal size, coating coverage, and pressure drop of the treated filters were measured to find an appropriate coating method for the filter. For lower salt and surfactant loading (< 5 mg/cm^2^), the nebulization coating showed a more uniform coverage and a lower increase in the filter pressure drop. However, for higher loading, clusters formed on the foremost layer of the filter, increased the pressure drop sixfold. The crystal size for the nebulized coatings ranged from 20 nm to 1 µm, while the maximum crystal size observed for the drop-cast coating was greater than 500 µm.

The antiviral properties of the coating compositions were measured using plaque assays of Phi6 bacteriophage with *P. syringae* as the host. The maximum antiviral property was observed for NaCl- Tween 20 coated samples, which inactivated 10^8^ PFU of virus in 2 h on a single-layer filter. A single-layer Tween 20 filter with loading of 1 mg/cm^2^ can reduce the viral activity of 10^8^ PFU of virus from 10^9^ to 10^2^ PFU/mL in 2 h and the NaCl-coated filter can reduce viral activity of 10^8^ PFU of virus not more than from 10^9^ to 10^5^ PFU/mL in even in a high concentration. The dominant antiviral activity can be associated with the detergent properties of Tween 20, and NaCl was shown to have a complementary effect. Therefore, NaCl- Tween 20 coated filters have better antiviral activity but when it comes to filtration application and facemask, pressure drop is also important; and Tween 20 coating with loading of higher than 2 mg/cm^2^ can be considered as the most practical choice. All in all, the high antiviral properties of Tween 20 along with the negligible effect on the pressure drop across the coated filter make Tween 20 a suitable antiviral agent to be used on the surface of face masks and ventilation filters.

## Materials and methods

### Filters

**Figure 7:**
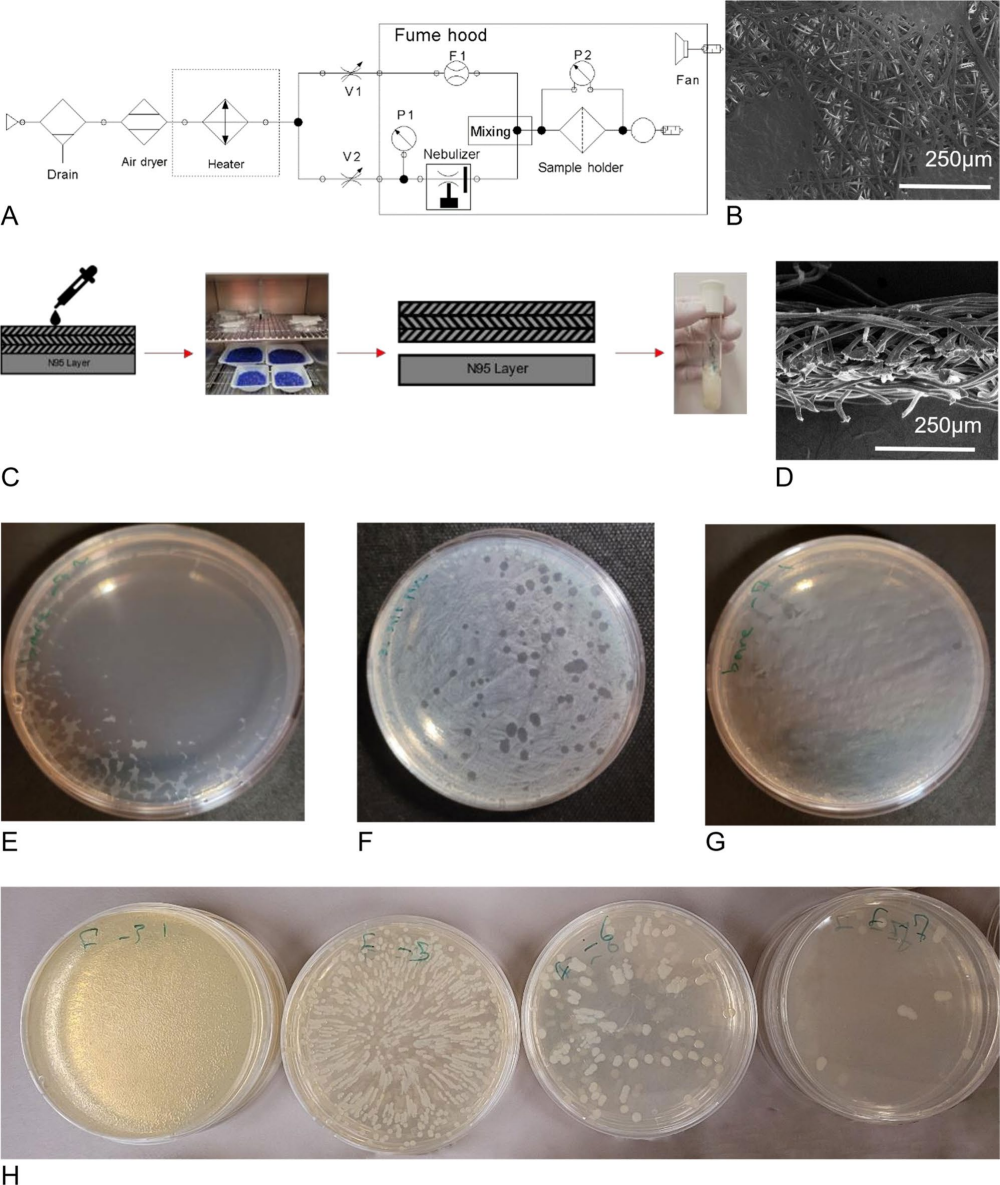
(**A**) A cross-sectional view of an uncoated filter; (**B**) The top view of an uncoated filter; (**C**) The testbed schematic used for coating and pressure drop experiments; (**D**) The procedure used to perform filter-involved experiments; (**E**) A fully infected Petri dish; (**F**) Discrete plaques are formed on surface of the Petri dish; (**G**) A non-infected Petri dish; (**H**) The assay of the bacterial stability experiment.

Spun bond single-use filters (Veratex polypropylene MF 150–50, Toronto, Canada) were used as the filter layer. The filters were cut into square shape pieces (30 mm × 30 mm) with scissors, then a 25.4 mm circular disk was taken out from the filters using a punch. All of the samples were labeled with a number, weighed, and placed in a ziploc bag for future use, Fig. 7B,D.

### Coating

The coating was performed based on three methods: dip coating, drop-cast coating, and nebulization.

#### Dip and rinsing coating

Dip coating was performed using 25% w/v NaCl (Fisher brand, USA) in deionized (DI) water. Due to the hydrophobic properties of the sublayer filter, 0.5% v/v polyethylene glycol sorbitan monolaurate surfactant (Tween 20, Sigma Aldrich) was added to the solution to reduce the surface tension and make the solution penetrate the filter^8,10,13^. The solution was then filter sterilized using a 0.2 µm filter. For dip coating, the filters were submerged in the fluid for 1 min and then transferred to Petri dishes to dry. For drop-cast coating, the filters were placed in Petri dishes and the required volumes (100 µL, 200 µL, 300 µL, 400 µL) of the solution were transferred and spread onto the filters using a micropipette. All of the samples were dried at room temperature overnight in a container with HEPA filtered ventilation. After drying, the mass change was measured and the filters were kept in sealed containers.

#### Nebulization coating

The nebulization coating was performed using the setup shown in Fig. 7A. The setup contained a compressed air supply line and an air dryer followed by a bifurcation and two valves: one having an absolute pressure gauge and the other having a flowmeter.

The line with a pressure gauge on it leads to the nebulizer (CompAir Compressor Tabletop Nebulizer System NE-C801, OMRON Healthcare, Inc., Japan), which was designated to work in 10 PSI with an average droplet particle size of 3 µm. The flow rate of the coating solution in the designated pressure is 0.33 mL/min and five volumes of the solution were tested (1 mL, 1.5 mL, 2 mL, 2.5 mL, 3 mL). Due to hydrophobic properties of filters, the coating with just the NaCl solution needs fine particles. To reach the submicron particle sizes and coat the filter deeply, 1.5% w/v NaCl in a DI water solution was used as the coating solution. The low concentration in the fluid resulted in nano particle-sized coating on the fiber’s surface and the uniform coating even in the inner layers of the filter. Also, to keep the coating method consistent for the biological experiment, the Tween 20 0.5% v/v and the NaCl- Tween 20 (25% w/v–0.5% v/v)-coated samples were coated using this mechanism. To ensure that there wasn’t any biological contamination and debris in the solution, the coating solution was filtered using a 0.2 µm filter.

There was a free airstream line in the coating apparatus that enhanced the evaporation of the water in the droplet and prevented the filter from getting wet and helped to make more uniform particles and reach smaller particle sizes. The free airstream was equipped with an air flowmeter (LZQ-5 2.5–25 LPM air flowmeter. Hilitand, China) to adjust the airstream to maintain the free airstream ratio as high as possible while keeping the filter face velocity as suggested by the manufacturer and preventing the particles from penetrating through the filter (0.5 m/s).

The two airstreams were then combined in a mixer. The mixer should have sufficient volume to give the droplets enough time to evaporate. For this experiment, the total flow rate of the airstream was kept under 15 Lpm (8 Lpm from the nebulized line and 7 Lpm from the free airstream). The mixer is a 20 cm cylindrical tube with a diameter of 15 cm placed vertically. The rapid increase in the cross-section resulted in rapid velocity reduction followed by the settlement of large particles which leads to only airborne particles reaching the filter.

The airstream then will be conducted to the filter holder (polypropylene filter holder for 25 mm membranes, Cole Parmer, USA). The active coating area for the filters is a 25 mm-circular surface. Due to the risk of fine particle spreading, the aerosolization apparatus was kept in a fume hood.

### Pressure drop measurement

Pressure drop measurements were conducted based on European standards (ES14683-2019) and American Society for Testing Standards (ASTM 2100–19) using the same apparatus for the nebulized coating with a closed nebulization line. For the pressure drop measurement, the flowmeter reads the total flow rate and a differential pressure transducer (vera low differential pressure transducer, Model 267, Setra Systems, Inc., USA) was used to calculate the pressure drop. The humidity and temperature were calculated at the exhaust of the system. The data from the temperature, humidity, and pressure drop transducers was collected using a DAQ System (National Instrument, USA) and a lab-made LabVIEW code.

### SEM microscopy

Scanning electron microscope (SEM) microscopy (FEI Helios, ThermoFisher Scientific Inc., USA) was performed at SFU’s 4D LABS. The samples were cut and placed on conductive carbon adhesive and sent to SFU 4D LABS in closed containers. The samples were coated with iridium to make a conductive layer for the microscopy. The photos used for the crystal size measurement were taken from the innermost layer of several cuts of random locations of the filters.

### Crystal size measurement

Two methods were used for crystal size measurement.

#### Dip coating and drop-cast coating crystal size measurement

The dip-coated samples' crystals size was measured using a digital microscope (AM7915MZTL, Dino Light, USA) and the crystal size was measured using ImageJ software. The surface area of each crystal was measured based on the pixels and using the conversion factor, the total surface area of each crystal was calculated. For each volume of transferred fluid, three samples were subjected to crystal size measurement.

#### Nebulization coating crystal size measurement

The crystal size of samples subjected to a nebulized coating was smaller and could not be measured using the previous method. SEM microscopy was used to depict the crystals, and the average crystal size and the coverage surface area were calculated using ImageJ software.

### Biological experiments

To evaluate the antiviral properties of the coating material, the plaque assay method was used. The method was used based on the methodology presented by Louis et al.^29^. Phages and bacterial host strains were obtained from the Félix d'Hérelle Reference Center for Bacterial Viruses of the Université Laval, QC, Canada^19^. The virus strain was (Phi6 Bacteriophage, HER 102, GREB, (Pavillon de Médecine Dentaire Université Laval, QC, Canada). The bacterial host was *Pseudomonas syringae* (HER 1102, GREB, Pavillon de Médecine Dentaire Université Laval, QC, Canada).

#### Agar preparation

King’s Agar^30^ was used for the biological experiments (1 L DI water, 10 g proteose peptone #2 (DIFCO), 1.5 g anhydrous K_2_HPO_4_, 15 g glycerol, and 15 g Bacto agar) then the solution was autoclaved at 121 °C for 20 min. The solution cooled down to 50 °C, then 5 mL of 0.2 µm filter-sterilized 1 M MgSO4 solution was added to the solution. Fifteen milliliters of the solution were transferred to the sterile Petri dishes using an autoclaved pipette. After cooling the solution to room temperature, the Petri dishes were placed in the fridge in closed bags to keep the moisture out and prevent contamination for future use.

#### Broth preparation

King’s Broth^30^ was used to culture bacteria and viruses. The procedure was the same with agar except without the Bacto Agar.

#### Phage amplification

A scratch of bacterial stock was inoculated with 5 mL KB broth and incubated in a water bath at 28 °C overnight to reach the OD_600nm_ of 2–3. Then, 100 µL of the bacterial culture was transferred to 5 mL of KB mixed with 100µL of phage lysate and incubated for 5–7 h at 28 °C. After that, the solution was filtered with a 0.45 µm filter and stored at 4 °C for future use. The viral activity using this method will be in the range of 5 × 10^8^ to 5 × 10^9^ PFU/mL.

#### Bacterial culture

To culture, the bacteria, a scratch of *Pseudomonas* was inoculated in 2 mL of KB and incubated overnight in a shaking water bath to reach an optical density (OD)_600 nm_ of 2. Then,100 µL of the bacterial culture was added to 2 mL of KB to reach an OD_600nm_ of 0.1 and placed in water bath for 4–5 h to reach the log phase (OD_600nm_ of 0.5–0.7). Fifty milliliters of the bacterial culture were transferred to the agar-loaded Petri dishes and spread with autoclaved glass beads.

#### Filter preparation

The filters were coated and weighed and then kept in a closed container to prevent contamination. On the day of the biological experiment, the filters were subjected to 2 W UVC radiation at 260–280 nm for 8 min to remove any contamination on them and then transferred to sterile Petri dishes.

#### Virus deposition on the filters

The virus (in the KB) was delivered to the filters using a P10 micropipette and based on the activity of each batch of virus culture a corresponding volume of 1–5 × 10^8^ PFU of the virus was delivered to the filter which is 50–250 µL in volume of the virus solution. The viral solution for control of each experiment was the same for all the other samples. The virus-loaded filters were placed in an incubator at 30 °C. To enhance the evaporation of the droplets, the bottom shelf of the incubator was loaded with desiccant beads (Blue desiccant beads). The filters were stored in the incubator three times (1, 2, and 4 h) for virus time stability, and 2 h for all other filter-involved experiments, Fig. 7C.

#### Virus washing

To evaluate the virus activity on filters, the viruses must be washed out from the filters and then the resulting solution will be subjected to serial dilution for plaque assays. To do so, right after the incubation, the filters were transferred to a sterile test tube and 5 mL of the KB solution was poured on them. The virus-containing test tubes were then placed on a shaker at maximum speed for 20 min, followed by an additional hour to rest both at room temperature. The resulting solution was then subjected to serial dilution and a plaque assay. The reported viral activity for the filters is the viral activity of the solution resulting from this step. (Fig. 7C).

#### Plaque assay

To evaluate the viral activity of each sample, the sample was serially diluted with a factor of 10 with KB broth. Fifty microliters of each dilution were then be transferred to the bacterial lawns grown in the Petri dishes and spread by glass beads. After taking the glass beads off of the Petri dishes, the Petri dishes were placed upside down in an incubator overnight at 27 °C. After 24 h. of incubation, the formation of plaques, (dishes with 15–150 plaques), Fig. 7E,F,G were counted to evaluate the viral activity of the 50 µL of sample. The viral activity of each sample was reported based on the activity of the undiluted viral solution using the following equation:

$$\frac{PFU}{ml}=\frac{Average number of plaques\times 20}{Dilution factor}$$

where PFU stands for Plaque Forming Units and the average number of plaques were determined from 3 technical replicates for one independent biological replicate. The results were from at least three biological replicates that were performed on separate dates.

A negative control (a sample with no bacteriophage) and positive control (a sample with bacteriophage) were included in all of the experiments. The positive control was the pure active virus subjected to a plaque assay and the negative control was the bacteria transferred to a Petri dish with 50 µL of KB instead of the virus solution.

### Statistical analysis

To reduce the risk of errors in the experiments, the biological experiments were done in triplicate technical replication and two biological replications, and the range of results was presented in error bars. The coating loading measurements and surface coverage analysis were done in quadruplets. The Single-factor Analysis of Variance (ANOVA) was performed for experiments and F and P-values for each experiment are reported. For penetration experiments, two by two comparisons were performed by Student T-test and t-value and p-value were reported. Because the variation in the activity of viruses and bacteria is logarithmic, the statistical tests were performed on the logarithm of the data. To calculate the Student t-test and ANOVA the critical p-value for both tests was p < 0.05(Microsoft Excel 2019 data analysis toolbar).

### Bacterial and viral stability experiments

The coated material on the filter is soluble in water, therefore there must be validation that the chemicals do not affect the bacterial activity. To do so, bacterial stability and viral stability experiments were performed. The bacterial stability experiments were done to make sure the bacterial activity remains constant in the presence of the chemicals, and the viral stability experiments were done to check the same for viruses.

#### Bacterial stability

A batch of overnight cultured bacteria was subjected to serial dilution to the factor of 10 and then transferred to the Petri dishes using the procedure stated earlier. Then a KB solution was made with an additional 1.5% w/v NaCl to simulate the maximum concentration of additional salt resulting from the coating to the washing solution for the NaCl coated filters. To simulate the maximum surfactant, the same procedure that was done for 0.03% v/v of Tween 20 was made and for NaCl-Tween 20 KB mixed with 1.5% w/v NaCl and 0.03% v/v Tween 20. The concentration of Tween 20 was kept low to be close to the critical micelle concentration. To perform each experiment, 50 µL of each solution was added to the Petri dishes loaded with bacteria. The negative and positive controls were also in the experiment. The resulting assay is shown in Fig. 7H.

#### Virus stability

The virus stability experiments are more critical because the virus will be subjected to extreme conditions (more than bacteria); hence, the virus stability experiments are divided into two experiments: the stability of the virus in the washing solution and the stability of the virus in the incubator.

1. For the washing solution stability, 5 × 10^8^ PFU of the active virus were added to 5 mL of KB and the same procedure for the washing was simulated and the virus activity was measured. This procedure was repeated for 3 concentrations of NaCl (0.5% w/v, 1% w/v, and 1.5% w/v), Tween 20 (0.01% v/v, 0.02% v/v, and 0.03% v/v), and NaCl-Tween 20 (0.5%w/v-0.01% v/v, and 1% w/v-0.02% v/v, 1.5% w/v-0.03% v/v) with positive and negative controls for each experiment. After adding the virus to the solutions, the samples were placed in a shaker at a maximum speed for 20 min, rested at room temperature for one hour, and then subjected to a plaque assay.
2. To evaluate the virus stability in the incubator, 5 × 10^8^ PFU of active virus in 10 µL droplets were transferred to both coated and uncoated filters, then the viral activity was measured during three different incubation times (1 h, 2 h, and 4 h). After the filter washing procedure, the viral activity was calculated. The results were compared with the pure active uncoated virus with positive and negative control.

## Data Availability

The datasets generated and analysed during the current study are available from the corresponding author and Milad Ebadi on reasonable request.
